# Convulsions, Their Pathology and Treatment

**Published:** 1858-06

**Authors:** B. M. Wible

**Affiliations:** Louisville, Ky.


### DEPARTMENT OF SELECTIONS.

### CONVULSIONS: THEIR PATHOLOGY AND TREATMENT.

*Founded mainly upon the teachings of the late Dr. Marshall Hall.* By B. M. Wible, of Louisville, Ky.

The frequency and fatality of convulsive affections, especially among children, render the subject an exceedingly interesting one to practitioners of medicine. Besides the accurate reports made in other countries, those derived weekly from some of our larger cities show how frequent and how fatal are the diseases of this character; and there are few physicians, I am sure, who have not seen enough to be impressed with their importance.

It appears to me that the pathology and treatment of this class of diseases, as taught in our standard books, have not been presented in a manner to accord with the present state of physiological and pathological science; and the object of this paper is to controvert some of the views held, and to present those which I think to be true. A leading error, which pervades almost all the dissertations I find upon this subject, consists in regarding convulsions as dependent, necessarily, upon a morbid condition of the brain. This idea has become so thoroughly interwoven with the views commonly entertained, that all the demonstrative teachings concerning the offices performed by the various parts of the nervous system have not been sufficient to dispel it from the minds of our most modern and able medical writers. Dr. West, without clearly defining the nervous centres involved in the convulsive phenomena, leads the reader to look upon the brain as the part chiefly at fault. He thinks congestion of this organ is generally a primary condition, and in some cases, when this view is out of the question, he substitutes for the congestion *cerebral irritation*. But it is difficult to define what are Dr. West's views of the pathology of convulsions as expressed in his *Lectures upon the Diseases of Children*. Dr. Wood, in his treatise upon the *Practice of Medicine*, holds similar views, and they are equally indefinite. In the *Cyclopedia of Practical Medicine*, Dr. Crawford and Locock hold to the leading error of the primary involvement of the brain, but it is candidly acknowledged by them that the essential condition of the nervous system in the disease is not understood. Dr. Cullen advanced the doctrine that convulsions were always the result of irritation of the brain, and this great error still holds dominion over the medical mind.

In examining the various authorities it is seen how truly convulsions have been traced to their causes; but in the absence of a solution of their mode of action, the treatment has been necessarily empirical, and sometimes hurtful. That the nervous system is involved in the convulsive phenomena is susceptible of the clearest proof. If the nerves be divided supplying one of the extremities of an animal, in which artificial convulsions have been excited by strychnine, it will be found that the extremity thus deprived will not participate with the rest of the body in the convulsive action; and it is clear, from this fact alone, that it is necessary that the nerves should possess an unbroken connection with the muscles. I am not aware, however, that it has been controverted that the muscular activity witnessed in convulsions is called forth by the nerves in connection with their centres; but the question is, What are the nervous centres involved? If a frog or any cold-blooded animal, or a very young warm-blooded animal, as a kitten or puppy, be brought under the influence of strichnine, the slightest excitation of any part of the surface will produce convulsive action; and if the brain be now removed, excitation will be followed by the same effect. If the viscera of the thorax and abdomen be removed, and with them the ganglionic system of nerves, leaving only the spinal centres in connection by their nerves, with the muscles, excitation will still develop the muscular contractions; but if the medulla spinalis, with the medulla oblongata, be at any time destroyed, then excitation fails to induce muscular action. On the other hand, by way of cross experiment, the brain may be sliced away,—that is, the cerebral and cerebellar lobes, the corpora striata, and the thalami optici,—without exciting any convulsive movements, and nothing of the kind is produced until the region of the medulla oblongata is invaded. It may be stated, in addition, that children have been born without brains, and have died convulsed. The conclusion seems inevitable from these facts, that the nervous centres which supply the force which calls the muscles into action in convulsions, are the medulla spinalis and medulla oblongata, and not the brain, as is too generally supposed; and this is the conclusion adopted by physiologists, but not by writers generally upon practical medicine, or at most the latter have not employed it when treating upon convulsions.

Assuming, then, the spinal system to be the source of the nerve-force which calls the muscles into action in convulsions, I will inquire as to the peculiarities of this force.

One of the peculiarities of the nerve-force of the spinal system is, that to develop it requires *excitation;* and it was on this account that Dr. Marshall Hall applied to it the term "*excito motor.*" That eminent man maintained that the brain may manifest its force *spontaneously*, while that of the spinal system is only called forth by some *excitation.* Thus, if a snake be divided half way between the head and the tail, it will be found that the part with the head will move spontaneously—that is, it will avoid its enemy, and seek to escape—while the other part will show no evidence of intelligence or of spontaneous movements; but it will move actively if touched or excited; and it is found that if this part be placed upon some soft substance and so covered as to be free from excitation from surrounding objects, it will never move; it displays, then, muscular activity in no other way but in obedience to excitation. If merely the head of the animal be separated, the remaining part shows the same peculiarity. The conclusion, then, is that the spinal system calls the muscles into activity only when it has been subject to excitation.

Another peculiarity of this force is, that it is capable of augmentation and of diminution. Its augmentation is produced by muscular inactivity or quiescence, and is found in persons of sedentary habits, and in some animals after hybernation; also by the prolonged excitation or irritation of the extremities of the nerves, such as arise from primary dentition, from decayed teeth—producing disease of the gums—from worms in the alimentary canal, from prolonged indigestion, from disease of the neck of the uterus, and, indeed, from morbid action in any part of the body freely supplied with incident spinal nerves. Traumatic tetanus furnishes a marked example of the wonderful augmentation of the nerve-force. It is produced also by certain medical or poisonous agents, as strychnine, opium, and other substances; and by a morbid state of the blood as is found in many diseases. Diminution of the nerve-force is produced by prolonged muscular effort, by shock from great injuries, by over-stimulation, by certain diseases termed adynamic, by chloroform, and perhaps by some of the narcotic substances.

In connection with these considerations it may be well to remark, that the spinal nerve force is in a degree antagonized by volition. In infancy and childhood the volition is feeble, while the spinal functions are strongly performed; and, to some extent, it is on this account that convulsive diseases are so much more frequent at that period of life.

Having presented these peculiarities of the nerve-force, as far as my limits will allow, I will now proceed to state the manner by which it is impressed in convulsions.

1st. By irritating agents making an impression on the element of the nerves, the impression being sent along the nerves to the spinal centres and thence reflected to the muscles.

2d. By irritating agents, such as foreign substances, making an impression directly on the centres; inflammation, or its effects; and poisons, or morbid materials circulating in the blood exciting the centre directly; and

3d. By mental emotions, which are known to act on the spinal centres, causing them to send out motor impulses.

The causes of convulsions may be classified according to the three modes by which the nerve-force is capable of being excited into activity.

The causes which act upon the extremities of the nerves are almost innumerable, and I shall allude only to a few of the more prominent of them.

Besides the accidents to which a child is subject during the process of parturition, the exposure of it to the external world—to a temperature colder than that to which it has been accustomed, to substances harder than those with which it has been in previous contact, the new processes by which its nutrition is supplied, and by which its blood is oxidized, the peculiar delicacy of its organization, with a nervous system highly susceptible to impressions—renders the period of early life one of much liability to convulsive diseases.

At any age the most frequent causes are those which make their impressions upon the organs of digestion. These are primary dentition; decayed teeth, attended with inflammation of the gums; an overloaded stomach; irritating and indigestible substances in the stomach; acidity of the stomach and bowels; indigested food which has passed into the colon and rectum; irritating secretions lodged in these parts; and worms in the stomach and large intestines.

It is well known that when convulsions arise from intestinal irritation the cause is in the stomach, or in the large intestines, and this corresponds with the anatomical fact that these parts are largely supplied with spinal nerves, of which the small intestines receive few or none.

While the alimentary organs are, perhaps, most frequently the seat of the excitation which gives rise to convulsions, it must be borne in mind that these may be produced by excitation of any part of the body supplied with spinal incident nerves; as the meninges of the brain, the skin, the respiratory surfaces, the kidneys, uterus bladder and urethra, and the vagina, uterus and ovaries.

The causes which act centrically in producing convulsions are numerous. A prominent one is that of effusion within the cranium. The effused fluids affect compression of the medulla oblongata, and in this way excite convulsions. That convulsions are thus excited I think is fully proved by an experiment made by Mr. Lawrence upon a living child which was born with none of the bones of the cranium present except the sphenoid, and none of the encephalic nervous structures except the medulla oblongata. He was enabled to excite convulsions in the child by slight pressure upon the exposed medulla oblongata. Abercrombie relates a case of a child eight months old, in which, at an early period of its complaint, "there was observed a remarkable prominence of the anterior fontanelle," which increased to a circumscribed tumor, soft and fluctuating,—and pressure upon it occasioned convulsions.

Besides the causes which act mechanically on the nervous centres, various substances circulating in the blood may excite them and produce convulsions. In this way poisonous agents, absorbed in the circulation, produce their influence on the nervous system. The convulsions which so often occur during the eruptive fevers, as smallpox, scarlatina, and measles, and those which arise in the course of many other diseases, are no doubt owing to some material circulating in the blood and irritating the nervous centres.

The suppression of the urine, or the arrest of any habitual discharge, is apt to be followed, it is well known, by convulsions.

As a cause of convulsions the *emotions* must not be overlooked. The seat of the emotions is undoubtedly in the brain ; but they manifest their influence on the muscles independently of the will. The mode in which they are supposed to act is by an influence transmitted from the brain to the spinal centres; thence a motor influence is propagated to the muscles. Muscular contractions as observed to take place under the influence of any of the strong emotions; and in excitable persons, especially children, they often amount to convulsions. The liability of the emotions to excite convulsions will greatly depend upon the previous excitability of the nervous system. They are more liable to have this effect when they co-exist with any of the peripheral irritations, as that of dentition or gastro-intestinal disturbances ; and it is plainly to be observed how startings at such times are produced by the emotions.

The premonitory symptoms of a convulsive attack are to be found in the irregular actions of the motor apparatus in various parts of the body—as sudden startings, fixation or oscillation of the eyes, alternate contraction or dilation of the iris, the varied expression given by the muscles of the face, and the peculiar contractions of the hands and feet. When the convulsion has commenced, the muscles of all parts of the body are, in some cases, involved ; in others, only partially. The muscles of the neck are found rigid and jerking, twisting the head around upon the shoulder, and arresting the return of the venous blood from the head. The respiration previously irregular and sighing, is, during the attack, often suspended for a considerable time. The suspension of the breathing depends upon the fixation of the muscles of respiration and upon spasm of the glottis.

That I may not be misunderstood, I will make a brief statement as to the extent to which the *brain* is involved in the disease.

Convulsions may arise from inflammation of the meninges of the brain, or from spiculæ of bone pressing upon them, and thus exciting the terminal extremities of the nerves distributed to them. In these instances the convulsions are excited by eccentric, and not centric excitation, as is sometimes stated. The inflammation may, however, extend to the medulla oblongata, and produce direct or centric excitation; or, it may terminate in effusion, and effect counter pressure. The convulsions which occur toward the fatal termination of meningitis are generally the result of effusion. It is possible that great cerebral congestion, without effusion, may produce counter-pressure sufficient to excite convulsions, but this is hardly probable.

On the other hand the brain may be involved as an *effect* of the convulsions; the fixation of the muscles of respiration, and the spasmodic contractions of the muscles of the neck, retard the venus blood returning from the head, and produce passive congestion of the encephalon. This passive congestion may in turn, by counter-pressure, add to the severity of the paroxysm; and it is, moreover, the cause of the insensibility, coma, stupor, and mental aberration which accompany and succeed the convulsion.

While the brain, then, is sometimes, but not necessarily involved, it must be borne in mind that the centres essentially concerned are those of the excito-motory system.

The prognosis of an attack of convulsions may generally be regarded as favorable if the cause can be promptly removed; but death may result from embarrassment of the respiration—particularly from spasm of the glottis—in cases otherwise favorable. When the cause is some irritating substance in the stomach or large intestines, it may be properly removed by an emetic, or by injections into the lower bowels, and in such cases a favorable termination may be expected. When they come in the course of an eruptive fever, the development of the eruption generally terminates the convulsion. On the contrary, when they arise from effusion within the cranium, or from any persistent cause, the prognosis is unfavorable.

*Treatment.*—If the nature of convulsion is understood—that is, the *laws of action* of the nervous system, and the *causes* that affect it—the true plan of treatment can be readily deduced. Two leading indications are to be observed. The first is to remove the cause, and the second is to overcome the preternatural excitability of the excito-motory or spinal nervous system. If the cause be effusion within the cranium, the result of inflammation of the brain or its envelopes, but little can be anticipated from any plan of treatment that can be adopted. The remedies for cerebral inflammation, and those that promote the absorption of effused fluids, are the only ones that can be employed, except such as tend to allay the excitability of the nervous system. If the cause be either active or passive congestion of the brain, the moderate abstraction of blood, the elevation of the head and shoulders, continued cold applications to the scalp, the removal of every cause of eccentric irritation, and the avoidance of the emotions, together with such means as will be hereafter alluded to as calculated to reduce augmented excitability, will constitute the resources to be employed in the treatment. If the case be one of eruptive diseases, efforts ought to be made to promote the appearance of the eruption by the use of gentle stimulants, the employment of diluents, and such other means as conduce to effect that object. If the cause be the suspension of any of the secretions, or the stoppage of any habitual discharge, their restoration ought to be aimed at. If strychnia, opium, or any deleterious agent of the kind have been taken into the system, its ejection should be effected as far as possible, and such antidotes given as tend to overcome the effects of the poison. In cases where the cause is some unfriendly material circulating in the blood there is no direct or prompt way to get rid of it; then, besides the use of remedies which aid in its elimination, those calculated to soothe the too excitable nervous system should be resorted to, and those which excite it avoided.

The convulsions most frequently seen in practice are such as arise from unfriendly substances in the stomach, or in the large intestines; and the treatment of cases of this character is the most satisfactory. To remove the cause is the prime object. When indigestible food, or other irritating agents in the stomach, give rise to convulsions, prompt emetics, as of sulphate of zinc, or of ipecacuanha, should be directed. If the fluids of the stomach are acid, the bicarbonate of potassa may be advantageously given with the emetic. After the emetic has acted, some mild alkaline drink ought to be administered, to correct the acidity which is apt to follow the irritation of the stomach. When the convulsions arise from the irritating accumulations in the large intestines, I know of no remedy which so readily relieves as the free injection of warm water into the bowels. This treatment I have pursued for several years, and it has been most satisfactory. Recently, an article appeared in the *London Lancet*, commending in the highest terms this simple treatment; and the explanation given of its efficacy was that the warm water distended and soothed the irritated bowel, prevented it from contracting upon its irritating contents, and promote the expulsion of them. Certainly this treatment is more efficacious and rational than the one generally advised, of giving stimulating injections to produce a repulsive effect.

When the convulsions are produced by irritants, or morbid action in any other part of the body supplied with incident nerves, as the gums, the urinary organs, or the uterus and vagina, the plan of treatment should consist in removing the cause by such means as are suggested by therapeutical principles. Frequently several causes are found to exist; thus, there may be a swollen state of the gums, while the digestive organs are loaded with irritating matter. In such cases the gums should be divided, and the digestive organs freed from whatever is irritating them.

The second indication—viz. the reduction of the augmented excitability of the nervous system—constitutes an important part in the treatment of convulsions. In the first place, besides removing the various causes which give rise to the excitable condition, the contravening of the various sources of excitation to the nervous system, and the avoidance of mental emotions, constitute an important part of the treatment.

In treating convulsions it is not always that the cause which gave rise to them can be at once removed; and while this is being attended to the warm water will have an excellent effect in quieting and soothing the prostrated nervous system; indeed it is the most effectual remedy to control excitability. It should be at the temperature of from 100 to 110°. It is probable the high temperature that controls the excitability, if some recent experiments made, I believe, by Edward, can be relied on.

When the patient is placed in the bath, the temperature should not be so high, otherwise the first contact with the bath will excite. After the patient is placed in the bath, at a temperature which neither gives a disagreeable sense of heat or cold, then hot water should be gradually added to attain the requisite temperature. After the patient is removed from the bath, friction to dry the surface must be prohibited. It is best to cover the patient comfortably in a bed, without attempting to dry the surface, as the friction necessary to accomplish this might continue or excite the convulsion. Baths of this kind ought to be repeated from time to time, as often as any threatenings remain of a return of the paroxysm. After the stomach and bowels have been freely evacuated, and after active cerebral congestion has been overcome by blood-letting, hyoscyamus has a decided effect in lessening the nervous excitability. Perhaps other articles, usually called antispasmodics, would have the same effect; but upon none of them do I place so much reliance as upon the hyoscyamus.

The loss of blood is always injurious. It increases the excitability, and in consequence of which, in any case, the loss should be moderate.

It will be inferred, from what I have stated, that all rude medication, as mustard-plasters, frictions of the skin, and irritating purgatives are positively injurious: they are advised, however, by our standard writers.

After a convulsive seizure has passed, it is the duty of the physician to prescribe such a course of medication and regimen as will obviate the liability to its return. For this purpose a regulated diet, attention to the digestive organs, cold bathing, free exercise in the open air, and such other means as will give vigor to the system, should be enjoined. In directing prophylactic or curative means, it must be borne in mind that the disease is one in which the nervous system is *excited* to action; therefore, all agents which would have that effect must be avoided.

In the imperfect outline which I have here attempted to draw of convulsions, the principles presented apply to the entire class of convulsive and spasmodic affections—as puerperal convulsions, epilepsy, apoplexy, chorea, etc. They apply also to many of the cases of functional and sympathetic derangements of the organs of the body. Convulsions are essentially related to the spinal nervous system—a system that holds extensive relations in the animal economy, and plays an important part in almost every disease.

